# High-throughput RNA-sequencing identifies mesenchymal stem cell-induced immunological signature in a rat model of corneal allograft rejection

**DOI:** 10.1371/journal.pone.0222515

**Published:** 2019-09-23

**Authors:** Xiaoxiao Lu, Chenchen Chu, Xun Liu, Yichen Gao, Mianmian Wu, Fang Guo, Yahong Li, Chao Geng, Yue Huang, Yan Zhang, Shaozhen Zhao

**Affiliations:** Tianjin Key Laboratory of Retinal Functions and Diseases, Eye Institute and School of Optometry, Tianjin Medical University Eye Hospital, Tianjin, China; Children's Hospital Boston, UNITED STATES

## Abstract

**Objective:**

The immune rejection mediated by CD4^+^ T cell and antigen presenting macrophages is the leading cause of corneal transplantation failure. Bone marrow-derived mesenchymal stem cells (BM-MSCs) possess robust immunomodulatory potentials, and have been shown by us and others to promote corneal allograft survival. However, the immunological mechanism underlying the protective effects of BM-MSCs remains unclear. Therefore, in the current study, this mechanism was investigated in a BM-MSC-treated rat model of corneal allograft rejection, in the hope to facilitate the search for novel interventional targets to corneal allograft rejection.

**Methods:**

Lewis rats were subjected to corneal transplantation and then received subconjunctival injections of BM-MSCs (2×10^6^ cells / 100 μl PBS) immediately and at day 3 post-transplantation. The control group received the injections of PBS with the same volume. The clinical parameters of the corneal allografts, including opacity, edema, and neovascularization, were regularly evaluated after transplantation. On day 10 post-transplantation, the corneal allografts were collected and subjected to flow cytometry and high-throughput RNA sequencing (RNA-seq). GO enrichment and KEGG pathways were analyzed. The quantitative realtime PCR (qPCR) and immunohistochemistry (IHC) were employed to validate the expression of the selected target genes at transcript and protein levels, respectively.

**Results:**

BM-MSC subconjunctival administration prolonged the corneal allograft survival, with reduced opacity, alleviated edema, and diminished neovascularization. Flow cytometry showed reduced CD4^+^ T cells and CD68^+^ macrophages as well as boosted regulatory T cells (Tregs) in the BM-MSC-treated corneal allografts as compared with the PBS-treated counterparts. Moreover, the RNA-seq and qPCR results demonstrated that the transcript abundance of *Cytotoxic T-Lymphocyte Associated Protein 4* (*Ctla4*), *Protein Tyrosine Phosphatase*, *Receptor Type C* (*Ptprc*), and *C-X-C Motif Chemokine Ligand 9* (*Cxcl9*) genes were increased in the allografts of BM-MSC group compared with PBS group; whereas the expression of *Heat Shock Protein Family A (Hsp70) Member 8* (*Hspa8*) gene was downregulated. The expression of these genes was confirmed by IHC at protein level.

**Conclusion:**

Subconjunctival injections of BM-MSCs promoted corneal allograft survival, reduced CD4^+^ and CD68^+^ cell infiltration, and enriched Treg population in the allografts. The BM-MSC-induced upregulation of *Ctla4*, *Ptprc*, *Cxcl9* genes and downregulation of *Hspa8* gene might contribute to the protective effects of BM-MSCs and subserve the potential interventional targets to corneal allograft rejection.

## Introduction

Corneal transplantation is the main therapeutic modality to corneal blindness[1]. Although corneal allografts, to certain extent, are protected by ocular immune privileges, immune rejection remains the leading cause of keratoplasty failure[2]. Accumulating evidences indicate that the immune responses mediated by CD4^+^ T cells and antigen presenting macrophages play critical roles in launching the immune rejection[3]. Currently in the clinics, the immunosuppressive agents, including Cyclosporin A, FK506, and glucocorticoids, can inhibit immune responses, subdue inflammatory cell infiltration, and prolong corneal allograft survival[4, 5]. However, long-term use of these immunosuppressives is associated with severe side effects, such as cataract and elevated intraocular pressure, which profoundly limit their clinical applications[6]. Therefore, a new, effective, and safe therapeutic modality is needed to inhibit immune rejection and promote corneal allograft survival.

The mesenchymal stem cells (MSCs) possess immunomodulatory and anti-inflammatory properties, self-renewal and multi-lineage differentiation potentials, and tissue repair functions[7–10]. During the last decade, research has shown that MSCs are able to modulate immune responses in autoimmune diseases, such as rheumatoid arthritis[11] and Type I diabetes[12, 13]. Importantly, the MSCs can also inhibit immune rejection during transplantation of vital organs, including skin[14], heart[15], islet[16], and kidney[17]. Furthermore, it has reported that MSCs, particularly bone marrow-derived MSCs (BM-MSCs) can promote survival of corneal allografts through antagonizing innate and adaptive immune responses, inhibiting activation and migration of antigen-presenting cells (APCs), and suppressing effector T cell functions[18–20]. Our recent studies showed that both systemic and local administration of BM-MSCs inhibited the immune rejection and significantly promoted corneal allograft survival[18, 19]. However, the immunological mechanism underlying the BM-MSCs’ protective effects on corneal allografts remains not completely understood.

With the development of new generation high-throughput sequencing technology, RNA sequencing (RNA-seq) has become a powerful tool to perform genome-wide transcriptional screening and compare differential gene expression profiling, thereby profoundly contributing to the search for molecular targets for novel interventional approaches[21–24]. In the present study, a rat model of corneal allograft rejection was established, and BM-MSCs or vehicle control phosphate buffered solution (PBS) were subconjunctivally injected to the rats with allografts. Flow cytometry and RNA-seq were employed to investigate the immunological mechanism underlying the BM-MSC’s protection of corneal allografts from immune rejection. An immunological signature induced by BM-MSCs and comprised of different proportions of infiltrating immune cells and differentially expressed genes was identified and validated in this study, shedding light on the interventional targets to corneal allograft rejection.

## Methods

### Animals

Female Wistar rats and Lewis rats (6–8 weeks of age, 180–200 g body weight) were purchased from Beijing Vital River Laboratory Animal Technology Co., Ltd. (Beijing, China). All the animals were housed under specific pathogen-free standard conditions under 12-h light–dark cyclic illumination and with the temperature of 25 ± 1°C and the relative humidity of 40–70% in the Animal Experimental Facility of the Eye Institute of Tianjin Medical University Eye Hospital. All rats were fed *ad lib* with food and water. All the experimental procedures were approved by the Institutional Animal Care and Use Committee of Tianjin Medical University (permission number: SYXK2009-0001) and adhered to the Guide for the Care and Use of Laboratory Animals published by the U.S. National Institutes of Health (NIH; Publication No. 85–23, revised 1996).

### BM-MSC culture

BM-MSCs from Wistar rats, termed as OriCell Wistar rat MSCs, were purchased from Cygen (Cat.# RAWMX-01001, Cyagen, Santa Clara, CA, USA) and cultured in 25 cm^2^ tissue culture flasks (Corning, Corning, NY, USA) at 37°C in a 5% CO2 incubator. OriCell MSC growth medium (Cat.# GUXMX-90011, Cyagen, Santa Clara, CA, USA), consisting of 88% OriCell MSC basal medium, 10% MSC-qualified fetal bovine serum, 1% penicillin-streptomycin and 1% Glutamine, were used for cell culture and changed every 2 or 3 d. BM-MSCs were trypsinized and passaged upon 80–90% confluence. BM-MSCs at passage 3 to 5 were used in the following experiments. Self-renewal and multi-lineage differentiation potentials of the BM-MSCs were verified in our previous studies[19].

### Corneal transplantation and treatment protocols

The rat model of corneal allograft rejection was established as previously described by the same experienced ophthalmologist[19]. Briefly, the donor Wistar rats were euthanized by intraperitoneal (IP) administration of an overdosed 10% chloral hydrate, and the grafts were immediately obtained from the right central corneas of the Wistar rats using a 3.5-mm-diameter trephine. The host Lewis rats were anesthetized with an IP injection of 10% chloral hydrate (0.3 ml / 0.1 kg), their right corneas were removed to expose the graft beds. The donor graft was placed onto the graft bed of Lewis rat’s, and immobilized with 8 interrupted 10–0 nylon sutures. Care was taken to protect the graft’s endothelia and the host’s lens and iris during the surgery and not to cause extra suffering of the animals.

The Lewis rats with the cornea allografts were randomly divided into BM-MSC and PBS groups (n = 35 / group). The BM-MSCs (2×10^6^ cells in 100 ul PBS) were subconjunctivally injected into the BM-MSC group of rats immediately (day 0) and at day 3 post-operation under the anesthesia of 10% chloral hydrate (0.3 ml / 0.1 kg). The PBS at the same volume was administered as a vehicle control into the corresponding group. The dosage and frequency of the injected MSCs were determined according to our prior study[19].

### Clinical assessment of corneal allografts

Clinical manifestations were monitored using a slit-lamp biomicroscope and anterior segment photographs taken by a Nikon D90 camera (Nikon Corporation, Tokyo, Japan) on a daily basis. The evaluating parameters of corneal allografts, including opacity (0–4), edema (0–2), and neovascularization (0–4) (Table 1), were used to calculate rejection index (RI) according to the previously described criteria[25]. When total scores of the evaluating parameters are ≥ 5 and corneal opacity was ≥ 3, the graft is deemed as rejected.

**Table 1:** The scoring system for clinical assessment of corneal allograft.

Opacity	0: complete transparent
	1: slight graft opacity
	2: moderate graft opacity, but iris texture visible
	3: severe graft opacity, but pupil visible
	4: complete opacity and pupil not visible
Edema	0: no edema
	1: moderate edema
	2: obvious edema with graft thickening
Vascularization	0: no vascularization
	1: new vessels growth to 25% of graft radius
	2: new vessels growth to 50% of graft radius
	3: new vessels growth to 75% of graft radius
	4: new vessels growth to center of graft

### Flow cytometry assay

The corneal allografts were carefully harvested, and the infiltration of lymphocytes and macrophages in the corneal allografts were analyzed using multi-parametric flow cytometry as previously described[26]. In detail, corneal allografts and corresponding corneal beds from PBS and BM-MSC groups (n = 5 / group) were carefully harvested at day 10 post keratoplasty, washed twice with PBS, minced, and digested in 50 ul Liberase TL (2.5 mg / ml; Sigma-Aldrich, St. Louis, MO, USA) for 30 min at 37°C following the method described elsewhere[27]. For detecting antigen expression on cell surface, the cell suspensions were filtered, washed, and stained with fluorochrome-conjugated antibodies to CD4 (APC-CD4, Biolegend, San Diego, CA, USA), CD8 (APC-CD8a, Biolegend, San Diego, CA, USA), and CD68 (FITC-CD68, Bio-Rad, Hercules, CA, USA) for 30 min at 4°C, the cells incubated with the corresponding IgGs were included as isotype contols. For detection of regulatory T cells (Tregs), the cells were incubated with APC-conjugated CD4 antibody as mentoned above, and then the cells were washed with Permealization Wash Buffer (Biolegend, San Diego, CA, USA), resuspended, and incubated with Fixation/Permealization Buffer (Biolegend, San Diego, CA, USA) at 4°C overnight. The next day, the cells were stained with Foxp3 (PE-Foxp3, Biolegend, San Diego, CA, USA) antibody according to manufacturer’s instruction, the isotype control of Foxp3 antibody was also included. Finally, the flow cytometry assay was performed using a FACSCalibur (BD Biosciences, San Jose, CA, USA), and the results were analyzed by FlowJo software (Ashland, OR, USA).

### High throughput RNA sequencing

At day 10 post-operation, the Lewis rats of both PBS and BM-MSC groups were euthanized by overdosed 10% chloral hydrate, the allografts and corresponding cornea beds (n = 9 /group) were carefully harvested, snap frozen in liquid nitrogen, and stored in -80°C; whereas the eyeballs of the rest of the animals were processed for paraffin sections and immunohistochemistry (IHC) (n = 6 / group).

Three harvested corneal allograft samples in each group were subjected to high-throughput RNA-seq by Novogene (Beijing, China). Specifically, total RNAs were isolated from the allograft samples, and the quality of the isolated RNAs was initially examined on 1% agarose gels. The RNA purity, concentration, and integrity were then assessed using the NanoPhotometer spectrophotometer (IMPLEN, CA, USA), Qubit RNA Assay Kit in Qubit 2.0 Flurometer (Life Technologies, CA, USA), and RNA Nano 6000 Assay Kit of the Bioanalyzer 2100 system (Agilent Technologies, CA, USA), respectively. Three micrograms of the total RNA from each sample was used as the input material. First, ribosomal RNA (rRNA) was removed by Epicentre Ribo-zero rRNA Removal Kit (Epicentre, USA). Then the sequencing libraries were generated using the rRNA-depleted RNA by NEBNext Ultra Directional RNA Library Prep Kit for Illumina (New England Biolabs, Ipswich, MA USA), the library fragments purified using AMPure XP system (Beckman Coulter, Beverly, USA). The library fragments were amplified through PCR using Phusion High-Fidelity DNA polymerase, Universal PCR primers, and Index (X) Primer following the treatment of USER Enzyme (New England Biolabs, Ipswich, MA USA). The library quality was assessed on the Agilent Bioanalyzer 2100 system, and the clustering of the index-coded samples was performed on a cBot Cluster Generation System using TruSeq PE Cluster Kit v3-cBot-HS (Illumia) according to the manufacturer’s instructions. Afterwards, the libraries were sequenced on an Illumina Hiseq 4000 platform with the depth of 40 times, and generated 12 gigabytes paired-end clean reads with 150 bp in length. The paired-end clean reads were aligned to the reference genome using HISAT2 v2.0.4. [28], assembled by StringTie (v1.3.1)[29] in a reference-based approach. Cuffdiff (v2.1.1) was used to calculate FPKMs of the mRNAs in each corneal allograft sample[30]. A model based on the negative binomial distribution was employed to determine the differential expression in the digital transcript expression data[30]. Transcripts with *P*-adjust less than 0.05 were designated as differentially expressed. The RNA-seq raw data are available at the NCBI Sequence Read Archive database (https://www.ncbi.nlm.nih.gov/sra/PRJNA551878) under the accession number (PRJNA551878).

Gene Ontology (GO) enrichment analysis of the differentially expressed genes were implemented by the GOseq R package. GO terms with the corrected *P* value less than 0.05 were considered significantly enriched by differential expressed genes. The statistical enrichment of the differentially expressed genes in KEGG pathways was analyzed by KOBAS software. The complete RNA-sequencing data were available at SRA database of NCBI.

### Quantitative real-time PCR

The collected corneal allograft samples (n = 6 / group) were subjected to quantitative real-time PCR (qPCR) to validate the RNA-sequencing results of the selected genes. Briefly, total RNAs from the grafts and corresponding cornea beds were extracted using Trizol reagent (Life Technologies, Grand Island, NY, USA) according to the manufacturer’s protocol. Reverse transcription kit (Fermentas, Waltham, MA, USA) was used to synthesize cDNA. SYBR Green Mix (Roche, Branford, CT, USA), DNA templates and specific primers (Table 2) were mixed, and the qPCR was performed using a HT79000 Real-Time PCR System (Applied Biosystem, Foster City, CA, USA). The program was comprised of 50°C for 2 min, 95°C for 10 min, followed by 40 cycles of 95°C for 15 s, 60°C for 1 min. The disassociation stage was added to check the amplicon specificity. *β-actin* was used as an endogenous reference gene. The relative mRNA expression levels of the target genes were analyzed using the 2^−ΔΔCt^ method.

**Table 2:** The PCR primers for qPCR.

Genes	Sequences
*Hspa8*-F	CACCGTGCCAGCTTACTTCA
*Hspa8*-R	CGTTGAGGCCAGCAATAGTTC
*Ctla4*-F	TCACCTGCAGCTGCCTTCTA
*Ctla4*-R	GCTTCAGAGAAGATTGGGATGAA
*Cd3d*-F	ACCCTGGCTGGTGTCATCA
*Cd3d*-R	AAAGCAGTAGACCCCCAAAGC
*Cd3z*-F	TCAGCAGGAGTGCAGATGCT
*Cd3z*-R	CGCCCTAGATTGAGCTCGTT
*Ptprc*-F	CCAATGTCAGCACCACAGATATC
*Ptprc*-R	TGCTTGCAAAGCCCAGAGT
*Cxcl9*-F	AATCAGCGATGCTCCTGCAT
*Cxcl9*-R	CTGTTTGAGGTCTTTGAGGGATTT
*β-actin*-F	TCTGTGTGGATTGGTGGCTCTA
*β-actin*-R	CTGCTTGCTGATCCACATCTG

### Immunohistochemistry

The IHC was also conducted to verify the expression of the selected genes at protein levels. In brief, the eyeballs from BM-MSC and PBS groups (n = 6 / group) were fixed in 10% natural formalin, paraffin-embedded, sagittally sectioned at 5 μm in thickness. The tissue sections were incubated with rabbit anti-rat CD3 antibody (ab5690, abcam, Cambridge, MA, USA), Ptprc antibody (ab10558, abcam, Cambridge, MA, USA), Cxcl9 antibody (ab202961, abcam, Cambridge, MA, USA), Hspa8 antibody (ab51052, abcam, Cambridge, MA, USA), and Ctla4 antibody (CD152, Thermo Fisher Scientific, Waltham, MA, USA) at 4°C overnight, respectively, per manufacturer’s protocols. Sections were then washed and then incubated with horseradish peroxidase-conjugated goat anti-rabbit secondary antibody (abcam, Cambridge, MA, USA) for 2 h at room temperature. Diaminobenzidine solution was used as a chromogen. The sections were counterstained with hematoxylin and observed under a BX51 microscope (Olympus Optical Co. Ltd., Tokyo, Japan). The pictures were taken using the cellSens Standard electronic system (Olympus Optical Co. Ltd., Tokyo, Japan) under identical optical parameters. The intensity of DAB immunostaining was quantified using Image J software (NIH, Bethesda, MD). Nine pictures covering every corneal section were analyzed, the computerized pixels for positive DAB staining in each picture were determined following the protocol described by Varghese et al[31].

### Statistical analysis

All data were presented as Mean ± SEM. Statistic Program for Social Sciences 20.0 (IBM SPSS Inc., New York, NY, USA) was used for statistical analysis. Kaplan-Meier method was used to compare the survival time of corneal allografts. The differences between BM-MSC and PBS groups were examined by *two-tailed unpaired t-test*. A *p* value less than 0.05 was considered significant.

## Results

### Subconjunctival injection of BM-MSCs promoted corneal allograft survival

**Fig 1:**
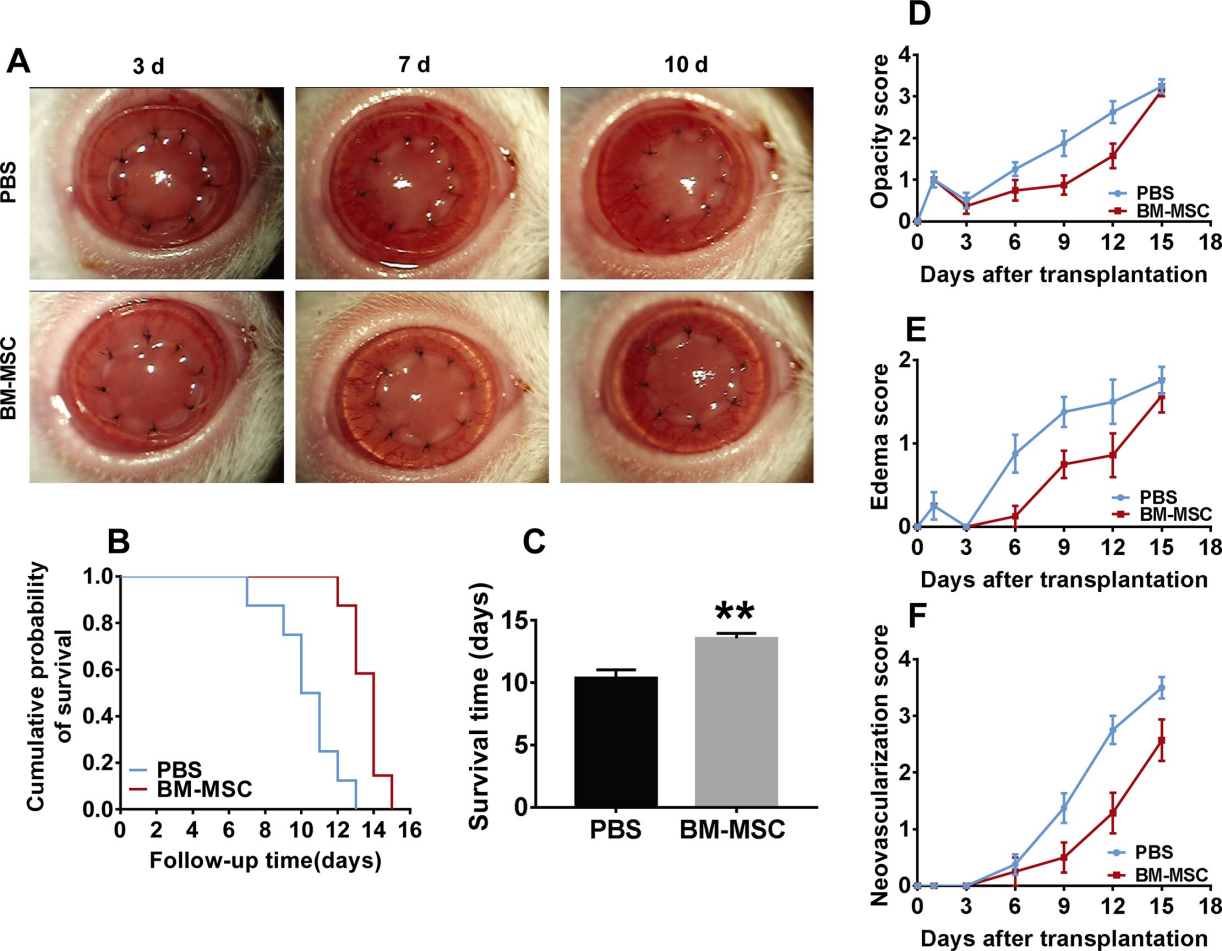
Subconjunctival injections of BM-MSCs prolonged corneal allograft survival with alleviated corneal opacity, edema and neovascularization. (A) Representative pictures of the corneal allografts from PBS- and BM-MSC-treated group at day 3, 7, and 10 post-operation. (B) Kaplan-Meier survival curve of corneal allografts (n = 8 / group). (C) Comparison of the mean survival time (MST) of the corneal allografts between the two groups (n = 8 / group). (D, E, F) The scores of opacity, edema, and neovascularization of the corneal allografts following penetrating keratoplasty.

To investigate the effects of BM-MSCs on corneal allografts, a rat model of corneal allograft rejection was established, and the BM-MSCs were subconjunctivally administered. As shown in Fig 1A, the corneal allografts in PBS group exhibited pronounced opacity and edema on postoperative day 7 and day 10 as compared to the allografts in BM-MSC group. Moreover, in the PBS group, there were more neo-vessels growing into the corneal grafts and reaching the graft center than the BM-MSC group at day 10 post-operation. Kaplan-Meier survival curve demonstrated that the mean survival time of the corneal allografts in PBS group was 10.38 ± 0.65 d, which was prolonged to 13.57 ± 0.37 d in BM-MSC group (Fig 1B), suggesting a modest but significant extension in the survival of the allografts treated with BM-MSC twice (Fig 1C, BM-MSC vs PBS, p = 0.0013). On the other hand, the corneal opacity, edema, and neovascularization were assessed on a daily basis under a slit-lamp microscope. The corneal allografts in both groups appeared slightly opaque and edematous on day 1 post-operation as a result of surgical trauma, which was alleviated on day 3 following operation. On day 6 and 9 post-operation, the corneal opacity, edema, and neo-vessels were significantly reduced in the grafts of BM-MSC group as compared with those of PBS group (Fig 1D–1F, all P < 0.05, BM-MSC vs PBS). These results demonstrated that subconjunctival injections of BM-MSCs subdued the corneal opacity, edema, and neo-vessel formation and promoted the survival of corneal allografts in the rat model of corneal allograft rejection.

### Flow cytometric analysis of the immune cells infiltrating in the corneal allgrafts

**Fig 2:**
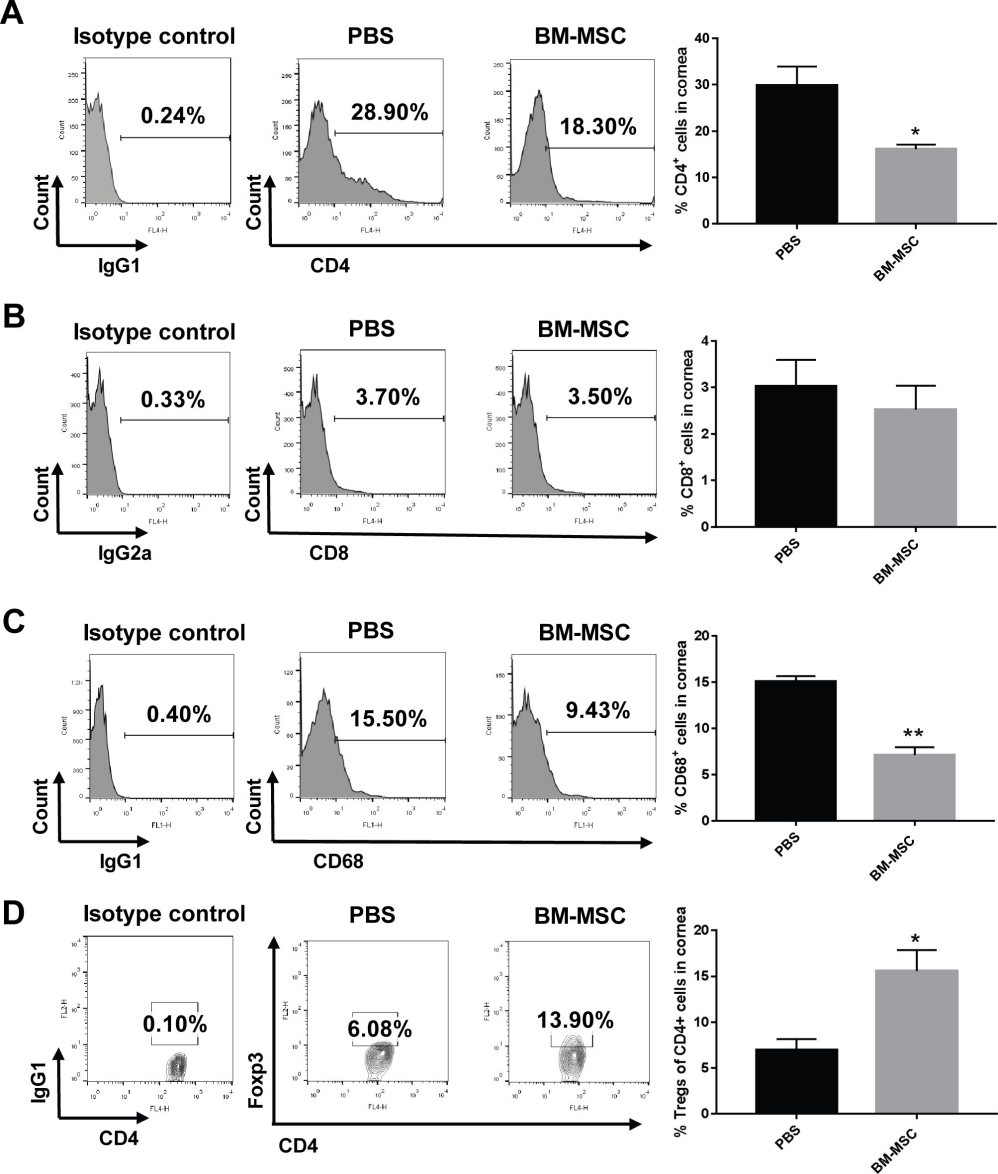
BM-MSCs administration inhibited CD4^+^ and CD68^+^ immune cell infiltration and augmented Treg proportion in the corneal allografts. Representative flow cytometric histograms and quantified bar graphs of CD4^+^ (A), CD8^+^ (B), and CD68^+^ (C) immune cells infiltrating in the corneal allografts on day 10 post-operation. (D) Representative flow cytometric contour plots and quantification of Foxp3^+^/CD4^+^ Tregs in the corneal allografts at day 10 following transplantation. n = 5 / group, * p < 0.05, ** p < 0.01.

Flow cytometry was employed to examine the immune cell infiltration at the site of corneal allografts. The percentage of CD4^+^ T cells infiltrating the allografts in PBS group was 29.88 ± 4.05%, which was significantly reduced to 16.16 ± 0.94% by the two subconjunctival injections of BM-MSCs (Fig 2A, P < 0.05, PBS vs BM-MSC). Furthermore, the frequency of CD68^+^ immune cells, indicative of antigen presenting macrophages, was reduced more than 50% in the BM-MSC-treated allografts as compared with that in the PBS-treated counterparts (Fig 2C, P < 0.01, PBS vs BM-MSC). By contrast, the frequency of CD8^+^ T cells in the two groups did not show any statistically significant difference, although a slight declination following the BM-MSC treatment was observed (Fig 2B, P = 0.537, PBS vs BM-MSC). More interestingly, the percentage of Foxp3+ and CD4^+^ cells, representing the Tregs, was boosted 2 fold after the BM-MSC treatment (Fig 2D, P < 0.05, PBS vsBM-MSC). These results suggest that local administration of BM-MSCs following corneal transplantation may reduce the infiltration of CD4^+^ T cells and CD68^+^ antigen presenting cells, as well as enrich the Treg population, implicating the possible cellular mechanism underlying the pro-survival effects of the locally-administered BM-MSCs.

### Gene expression profiling of the corneal allografts following local BM-MSC administration

**Fig 3:**
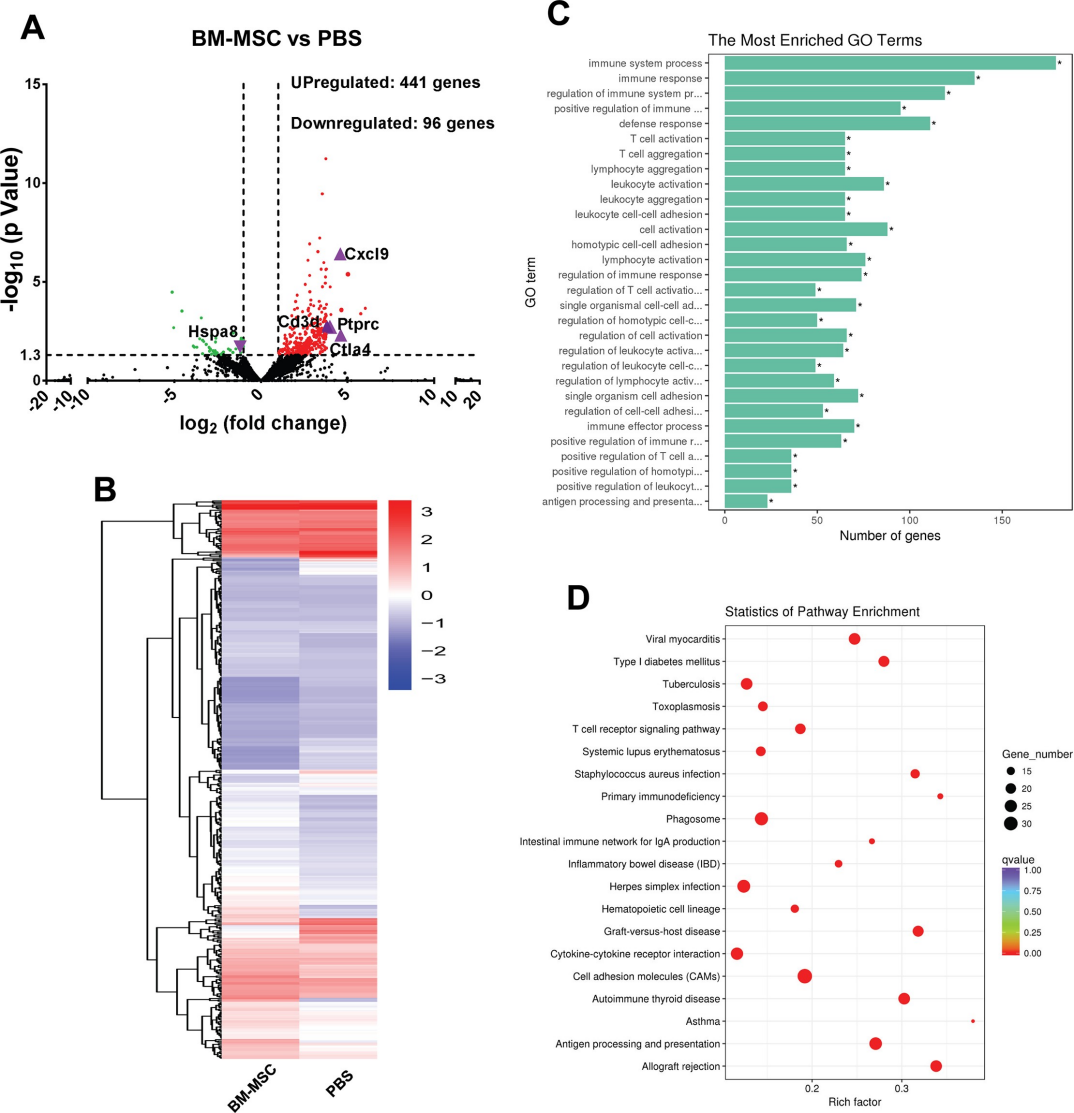
Comparasion of mRNA expression profiling between the corneal allografts in BM-MSC and PBS groups. (A) A volcano plot delineating the differentially expressed mRNAs between BM-MSC and PBS groups. The red dots represented significantly upregulated mRNAs and green dots downregulated (p < 0.05 with more than two fold absolute changes). The selected genes for validating gene expression profiles were marked by purple triangles. (B) Hierarchical clustering of mRNAs in PBS and BM-MSC groups (n = 3 / group). (C) GO analysis of the differentially expressed mRNAs. (D) The top 20 KEGG pathways for the differentially expressed mRNAs. GO, Gene Ontology; KEGG, Kyoto Encyclopedia of Genes and Genomes.

To identify the molecular immunological mechanism underlying the protective effects of the BM-MSCs on the corneal allografts, RNA-seq was carried out on day 10 post-transplantation and the mRNA expression profiles of the corneal allografts in BM-MSC and PBS groups were compared. Volcano plot delineated the differentially expressed genes between PBS and BM-MSC groups with P value less than 0.05 and absolute change more than two fold (Fig 3A). Hierarchical clustering heat map showed distinct gene expression patterns in the two groups (Fig 3B). Altogether, 537 mRNAs were differentially expressed in BM-MSC group as compared to PBS group, among which 441 upregulated and 96 downregulated. The top ten most upregulated or downregulated mRNAs were listed in Table 3.

**Table 3:** Differentially expressed mRNAs in corneal allografts and the related pathways.

Gene name	Fold changes	-log_10_(pvalue)	Regulation	Pathways
Col2a1	64.99649	3.659907	UP	Platelet activation, Amoebiasis, ECM-receptor interaction, Focal adhesion,PI3K-Akt signaling pathway
Slamf1	54.33613	3.389823	UP	Measles
Ccl5	32.64206	5.382971	UP	Herpes simplex infection, Cytokine-cytokine receptor interaction, Chemokine signaling pathway, Rheumatoid arthritis, Chagas disease (American trypanosomiasis), Prion diseases, Influenza A, Toll-like receptor signaling pathway, NOD-like receptor signaling pathway, Cytosolic DNA-sensing pathway, TNF signaling pathway
Sytl3	25.03683	3.5796	UP	--
Ctla4	24.49796	2.306209	UP	Autoimmune thyroid disease, Cell adhesion molecules (CAMs), T cell receptor signaling pathway, Rheumatoid arthritis
Cxcl9	23.85567	6.415331	UP	Cytokine-cytokine receptor interaction, Chemokine signaling pathway, Toll-like receptor signaling pathway
Cd247	16.43728	3.345338	UP	T cell receptor signaling pathway, Natural killer cell mediated cytotoxicity, Chagas disease (American trypanosomiasis)
Tnfsf14	15.97684	1.804369	UP	Herpes simplex infection, Cytokine-cytokine receptor interaction, NF-kappa B signaling pathway
Ptprc	15.70062	2.719765	UP	Cell adhesion molecules (CAMs), T cell receptor signaling pathway, Primary immunodeficiency, Fc gamma R-mediated phagocytosis
Cd3d	14.05869	2.75214	UP	T cell receptor signaling pathway, Primary immunodeficiency, Hematopoietic cell lineage, HTLV-I infection, Measles, Chagas disease (American trypanosomiasis)
FLOT2	11.6572	2.1253072	DOWN	Insulin signaling pathway
Krt76	10.32507	1.3602218	DOWN	--
Chrna5	10.32457	2.0483997	DOWN	Neuroactive ligand-receptor interaction
Nkain1	9.861725	1.9800326	DOWN	--
C2cd4c	7.978514	1.6805837	DOWN	--
Piezo2	7.830693	1.495555	DOWN	--
SH3BGR	7.510818	1.6031053	DOWN	--
A930017K11Rik	7.509152	1.602825	DOWN	--
Mrpl36	7.213453	2.2800434	DOWN	Ribosome
Hspa8	2.295773	1.7078015	DOWN	Antigen processing and presentation, Toxoplasmosis, Measles, Influenza A, Endocytosis, Epstein-Barr virus infection, Legionellosis, MAPK signaling pathway, Protein processing in endoplasmic reticulum, Estrogen signaling pathway, Spliceosome

### GO enrichment analysis of the differentially expressed genes between BM-MSC and PBS groups

A summary of GO enrichment analysis was shown in Fig 3C, suggesting that the differentially expressed genes between BM-MSC and PBS groups were mainly involved in the immune-related processes, such as immune system process, immune response, regulation of immune system process, T cell activation and aggregation, and antigen processing and presentation. This result implicates the immunomodulatory function of BM-MSCs in the model of corneal allograft rejection.

### KEGG pathway enrichment analysis

The pathway enrichment of the differentially expressed mRNAs was preformed based on the Kyoto Encyclopedia of Genes and Genomes (KEGG) pathways. As shown in Fig 3D, the majority of the differentially expressed mRNAs were enriched in “allograft rejection”, “cell adhesion molecules”, and “antigen processing and presentation”. The enrichment analysis in KEGG pathway showed that 970 genes were annotated, among which “cell adhesion molecules” (33 genes, 3.4%) was the most abundant, followed by “phagosome” (28 genes, 2.9%) and “antigen processing and presentation” (26 genes, 2.7%).

### Subconjunctival administration of BM-MSCs regulated the expression of heat stock protein, chemokines, and costimulatory molecule receptors

According to the GO enrichment and KEGG pathway analysis, six target mRNAs related to immune rejection, including *CD3d molecule (Cd3d)*, *CD3z molecule (Cd3z)*, *cytotoxic T-lymphocyte associated protein 4 (Ctla4)*, *protein tyrosine phosphatase receptor type C (Ptprc)*, *C-X-C motif chemokine ligand 9 (Cxcl9)*, and *heat shock protein family A (Hsp70) member 8 (Hspa8)* were selected from the top ten differentially expressed mRNAs as the “immunological signature” of the BM-MSCs. They could also serve as the candidate downstream molecules to relay or execute the protective functions of the BM-MSCs.

**Fig 4:**
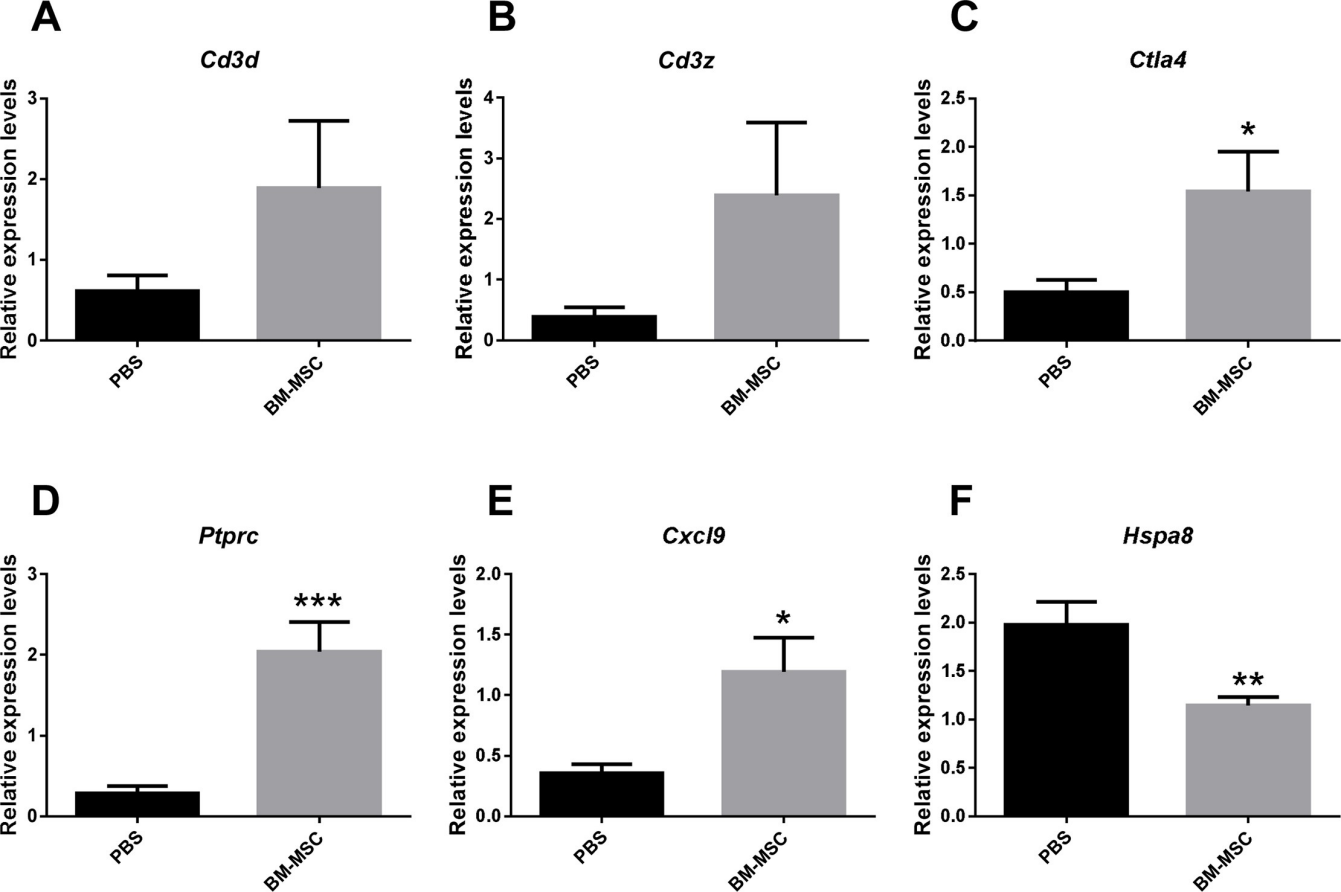
Validation of mRNA expression profiles by qRT-PCR. (A-F) The relative expression levels of the selected mRNAs (*Cd3d*, *Cd3z*, *Ctla4*, *Ptprc*, *Cxcl9*, and *Hspa8*) in the corneal allografts and recipient beds were determined by qPCR (n = 6 / group). *p < 0.05, **p < 0.01, ***p < 0.001.

The expression of these target genes were confirmed at the transcript levels by qPCR. As shown in Fig 4, the transcript levels of *Ctla4*, *Ptprc*, and *Cxcl9* genes were boosted more than 3 fold; whereas the level of Hspa8 mRNA was decreased 42% in the corneal allografts treated with BM-MSCs as compared with those in the grafts treated with PBS (All p < 0.05, BM-MSC vs PBS). These results were consistent with those from RNA-sequencing. The expression of *Cd3d* and *Cd3z* genes exhibited trendy upregulation after BM-MSCs treatments, however, the results were not significant (Fig 4, BM-MSC vs PBS, p = 0.138 for *Cd3d*; p = 0.100 for Cd3z).

**Fig 5:**
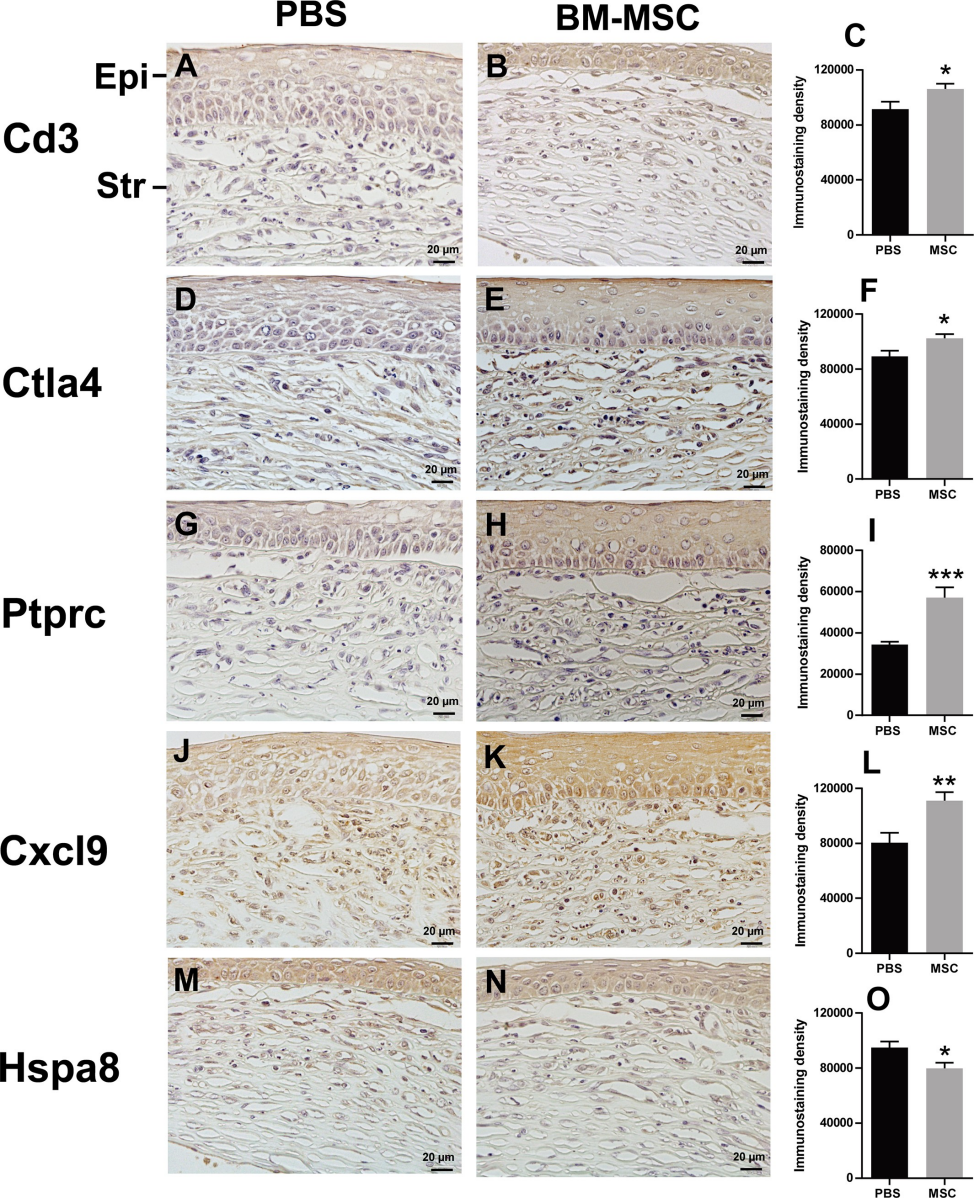
Validation of the gene expression at protein levels. Representative pictures of IHC staining and quantification of staining intensity of Cd3 (A-C), Ctla4 (D-F), Ptprc (G-I), Cxcl9 (J-L), and Hspa8 (M-O) in corneal allografts were shown. n = 6 / group. *p < 0.05, **p < 0.01, ***p < 0.001. Epi: epithelium, Str: stroma; Scale bar = 20 μm.

The IHC was also preformed to verify the expression of the selected genes at the protein levels. The expression trends of Ctla4, Ptprc, Cxcl9, and Hspa8 genes at the protein levels were similar to those at the transcript levels (Figs 4C–4F and 5D–5O), indicating that the expression of these genes were mainly regulated at the transcription level. It was notable that CD3 protein level in the BM-MSC group was significantly higher than that in the PBS group (Fig 5A–5C), which was in contrast to the nonsignificant upregulation of this gene at the mRNA level (Fig 4A and 4B). This result might reflect the differential regulatory mechanisms of this gene expression at transcript and protein levels.

## Discussion

This study demonstrated that subconjunctival injections of BM-MSCs prolonged corneal allograft survival, alleviated corneal opacity, edema, and neovascularization in a rat model of corneal allograft rejection model. Moreover, local administration of BM-MSCs reduced the infiltration of CD4^+^ T cells and CD68^+^ macrophages and increased the Treg population at the site of corneal allograft, hence indicating the cellular immunological mechanism underlying the BM-MSC’s pro-survival effects on the corneal allografts. Furthermore, high-throughput RNA-seq revealed the differential expression of *CD3*, *Ctla4*, *Ptprc*, *Cxcl9*, and *Hspa8* genes induced by locally-injected BM-MSCs. This gene expression profiling, validated by the conventional methods, for the first time explicitly links the immunomodulaotry property of BM-MSCs to the specific T cell receptor, chemokine, and costimulatory molecule receptor involved in immune rejection during corneal transplantation. Therefore, the infiltrating immune cells and the differentially expressed molecules constitute the unique immunological signature of BM-MSCs in the context of corneal allograft transplantation.

Cornea is an ideal tissue to investigate the effects of BM-MSCs on transplantation-induced immune rejection due to the easy access of target tissue and straightforwardness of clinical evaluations. For instance, corneal opacity, edema, and neovascularization are the three important parameters for evaluating corneal allograft status. Subconjunctival administration of BM-MSCs have been shown to mitigate these three parameters and promote the survival of corneal allografts. These results were consistent with the findings in the prior studies[18–20]. However, the exact molecular and cellular mechanism responsible for the protective effects of BM-MSCs has not been clarified.

Ctla4, also named as Cd152, is an inhibitory transmembrane receptor expressed on activated T cells[32]. Ctla4 induces an intrinsic T-cell inhibitory signal and negatively regulates immune responses through competitively binding to B7 molecules, such as CD80 and CD86, on APCs[32–35]. In addition, Sakurai et al[36] have discovered that Ctla4 is prone to promoting T cell differentiation into Th2 cells other than inducing immune tolerance. Therefore, Ctla4 may exert protection against immune rejection via inducing inhibitory T cell signaling, preventing T cell activation, and skewing the immune response to Th2-mediated response[32–35]. Indeed, several studies have used Ctla4-lg to inhibit or preclude T cell -mediated immune rejection after heart transplantation[34, 37, 38]. Our results confirmed the upregulation of *Ctla4* at both transcript and protein levels in the corneal allografts of BM-MSC group as compared to the PBS group, therefore, one may speculate that Ctla4 could be a downstream molecule mediating BM-MSCs’ suppressing immune rejection and promoting corneal allograft survival.

Ptprc, or CD45 antigen, is a type I transmembrane protein and functions as a key regulator of T and B cell antigen receptor signaling through co-stimulation with its extracellular domain or activating Src family kinases with its intracellular domain. In view of the flow cytometry data that Treg population was significantly enriched in the corneal allografts and the fact that Ptprc is expressed on Tregs[39], the upregulation of Ptprc following BM-MSC treatment could be, at least partially, due to the increased frequency of Tregs at the local environment of corneal allografts.

Cxcl9, also known as monokine-induced by interferon-γ, belongs to the CXC chemokine family. It is involved in Th1-type immune response under a variety of diseased conditions and activation of chemotactic lymphocytes[40]. Moreover, it has an extra capacity to inhibit neovascularization induced by chemokines, fibroblast growth factor, and vascular endothelial growth factor[41]. Vellasamy and colleagues[42] found that umbilical cord-derived MSCs elicit immunosuppression on activated T cells, at least in part, by downregulating the expression of *Cxcl9*, *IL-2*, *IL-2RA*, and *IFNG* genes. Qiao et al[43] also reported downregulation of Cxcl9 following transplantation of allogenic compact BM-MSCs for treatment of idiopathic pneumonia syndrome. By contrast, Xie et al[44] showed that coculture with BM-MSCs dramatically increased the expression of *Cxcl9* in hepatocellular carcinoma cells, implicating the possibility of Cxcl9 as a chemotactic factor for BM-MSC recruitment. Our result also showed the upregulated *Cxcl9* expression in corneal allografts that had been treated with BM-MSCs. The elevated levels of Cxcl9 protein might facilitate recruitment of BM-MSCs to the corneal transplantation site and subsequently contribute to anti-neovascularization.

Hspa8 is a heat shock protein that participates in numerous biological processes such as apoptosis, signal transduction, protein homeostasis, and autophagy[45, 46]. Hspa8 is also involved in activation of MAPK signaling during inflammation and infection[47, 48]. More importantly, heat shock proteins (HSPs) are recognized as potential target molecules for T-cell mediated immune rejection in heart and kidney transplants. Trieb et al[49] showed that the immune rejection to kidney allografts accompanied overexpression of HSPs, and the infiltrating monocytes in allograft transplantation site launched strong immune responses to Hspa8[49]. We detected significantly reduced mRNA and protein levels of *Hspa8* gene in the corneal allografts treated with BM-MSCs as compared to those treated with PBS, which might indicate the possibility that BM-MSCs could subdue the T cell-mediated immune response through downregulating the antigenic target.

In summary, subconjunctival administration of BM-MSCs inhibited immune rejection and promoted survival of corneal allografts in a well-recognized rat model of corneal allograft rejection. At the cellular level, BM-MSCs reduced the infiltration of CD4^+^ and CD68^+^ immune cells and enriched Treg population; at the molecular level, BM-MSCs local injections upregulated *Ctla4*, *Ptprc*, *Cxcl9* expression and downregulated *Hspa8* expression. The different proportions of immune cells and the differentially expressed genes generate an immunological signature of BM-MSCs, shedding lights on the potential interventional targets to corneal allograft rejection.
